# A comprehensive model of blood flow restriction in the postsurgical rat

**DOI:** 10.14814/phy2.70495

**Published:** 2025-08-05

**Authors:** Andin Fosam, Susana Castelo Branco Ramos Nakandakari, Isabella Chavez Miranda, Christopher Taber, Julie O'Connell, Andrew Raines, Yuichi Ohashi, Hualong Bai, Alan Dardik, Christina Allen, Rachel J. Perry

**Affiliations:** ^1^ Department of Internal Medicine, School of Medicine Yale University New Haven Connecticut USA; ^2^ Department of Cellular & Molecular Physiology School of Medicine Yale University New Haven Connecticut USA; ^3^ Department of Exercise Science Sacred Heart University Fairfield Connecticut USA; ^4^ Artelon Sandy Springs Georgia USA; ^5^ Vascular Biology and Therapeutics Program Yale University New Haven Connecticut USA; ^6^ Department of Orthopedic Surgery Yale University New Haven Connecticut USA

**Keywords:** ACL surgery, blood flow restriction, rat, resistance exercise ACL reconstruction

## Abstract

The mechanisms underlying blood flow restriction with low‐load exercise (BFR‐exercise)‐mediated muscle hypertrophy are not well understood. Lack of standardized rodent models of BFR‐exercise likely contributes to this gap. We demonstrate in male rats a comprehensive, clinically relevant protocol that generates a muscle loss state via bilateral anterior cruciate ligament reconstruction, achieves targeted, external blood flow occlusion, and performs weighted hind‐limb isolating knee extension exercise with BFR. These methods can be applied to mechanistic and physiologic studies of BFR‐exercise.

## 1 INTRODUCTION

Blood flow restriction (BFR) uses a temporary external occlusion cuff to partially restrict arterial blood flow and completely restrict venous outflow to and from distal muscles (Grant Mouser et al., 2018; Shinohara et al., 1998; Takano et al., 2005). When paired with lightweight exercise, BFR improves function in humans experiencing muscle loss from disparate etiologies (Abe et al., 2010; Iida et al., 2011; Jack et al., 2022; Jones et al., 2021; Kilgas et al., 2019; Kim et al., 2017; Patterson et al., 2019; Saatmann et al., 2021; Satoh, 2011; Takano et al., 2005; Telfer et al., 2021; Wilkinson et al., 2019; Yasuda et al., 2016). Despite promising outcomes, a better understanding of the underlying mechanisms will drive improved protocols for muscle loss. Several studies have used rats to understand the effects of aerobic and resistance exercise (Caponi et al., 2013; Emamian Rostami et al., 2021; Goutianos et al., 2015; Guzzoni et al., 2018; Kinney LaPier & Rodnick, 2001; Shi et al., 2018), but less so for BFR exercise (Gundermann et al., 2014; Nakajima et al., 2016; Takano et al., 2005).

In humans, quadriceps muscle loss in the operative limb can reach ~30%–40% within 4 weeks following anterior cruciate ligament reconstruction (ACL‐R) surgery (Lindström et al., 2013; Noehren et al., 2016; Norte et al., 2018; Setuain et al., 2017; Thomas et al., 2016). This can cause functional deficits and prolong recovery times (Keays et al., 2003; Kuenze et al., 2015; Lepley et al., 2020; Lepley & Palmieri‐Smith, 2015; Lisee et al., 2019). BFR‐exercise attenuates muscle loss following orthopedic surgeries, including ACL‐R (Colapietro et al., 2022; Jack et al., 2022; Kilgas et al., 2019), however rat models of ACL‐R primarily explore technical modifications and trial allografts of the ACL (Cardona‐Ramirez et al., 2022; Kaneguchi et al., 2023; Kawakami et al., 2021; Leong et al., 2015; Maurice et al., 2022). Transient BFR in rats is typically achieved in anesthetized rats using rubber bands and cuffs (Ramme et al., 2020; Song et al., 2023; Sudo et al., 2015; Tan et al., 2022; Yoshikawa et al., 2019), or permanent arterial occlusion via surgical ligation (Naderi‐Boldaji et al., 2019; Pour et al., 2017). External bands are simple and accessible; however, it is difficult to achieve replicable occlusion pressures. Cuffs generate more reproducible occlusions, but to our knowledge, have not been used to implement BFR in awake rats.

Resistance training in rats, tail‐weighted ladder climbing or incline treadmill walking (Ahtiainen et al., 2018; Al‐Sarraf & Mouihate, 2022; Guzzoni et al., 2018; Neto et al., 2016; Rodrigues Junior et al., 2023; Silvestre et al., 2017; Tan et al., 2022), employ similar muscle activation to traditional strength training (e.g., squats), but do not isolate hindlimb‐specific movement. This introduces compensation by the nonrestricted limbs. Preclinical studies combining BFR and resistance training use low‐current electrostimulation in anesthetized rats. This allows control of occlusion pressure but stimulates contraction of a single muscle (Garcia et al., 2022; Ramme et al., 2020; Yoshikawa et al., 2019) and does not reflect the contribution of synergistic muscles during resistance exercise.

The methods in this report lay a foundation for mechanistic studies of BFR‐exercise in rats. These studies will interrogate current mechanistic theories that debate the roles of circulating versus intramuscular metabolites, glycolytic muscle fiber shifts, and differences in mechanical work on the BFR‐mediated muscle hypertrophy.

## 2 METHODS

Procedures were approved by the Yale IACUC (protocol #20290). Male Sprague–Dawley rats (6–10 weeks) were housed in light‐ and temperature‐regulated facilities and fed a standard chow diet (Envigo Teklad #2018). The following protocols detail each methodological component.

### 2.1 Bilateral ACL‐R

#### 2.1.1 Graft preparation

1. Trim the 0.3 mm thick FlexBand Plus co‐polymer graft (Artelon, Sandy Springs, GA) to 3.0–3.5 cm (length) × 1.0–1.5 mm (width).
2. Attach a 5‐0 Vicryl or PGA suture to each end of the trimmed graft using a Krackow stitch, leaving 7–9‐cm suture tails.

#### 2.1.2 Surgical protocol

**FIGURE 1:**
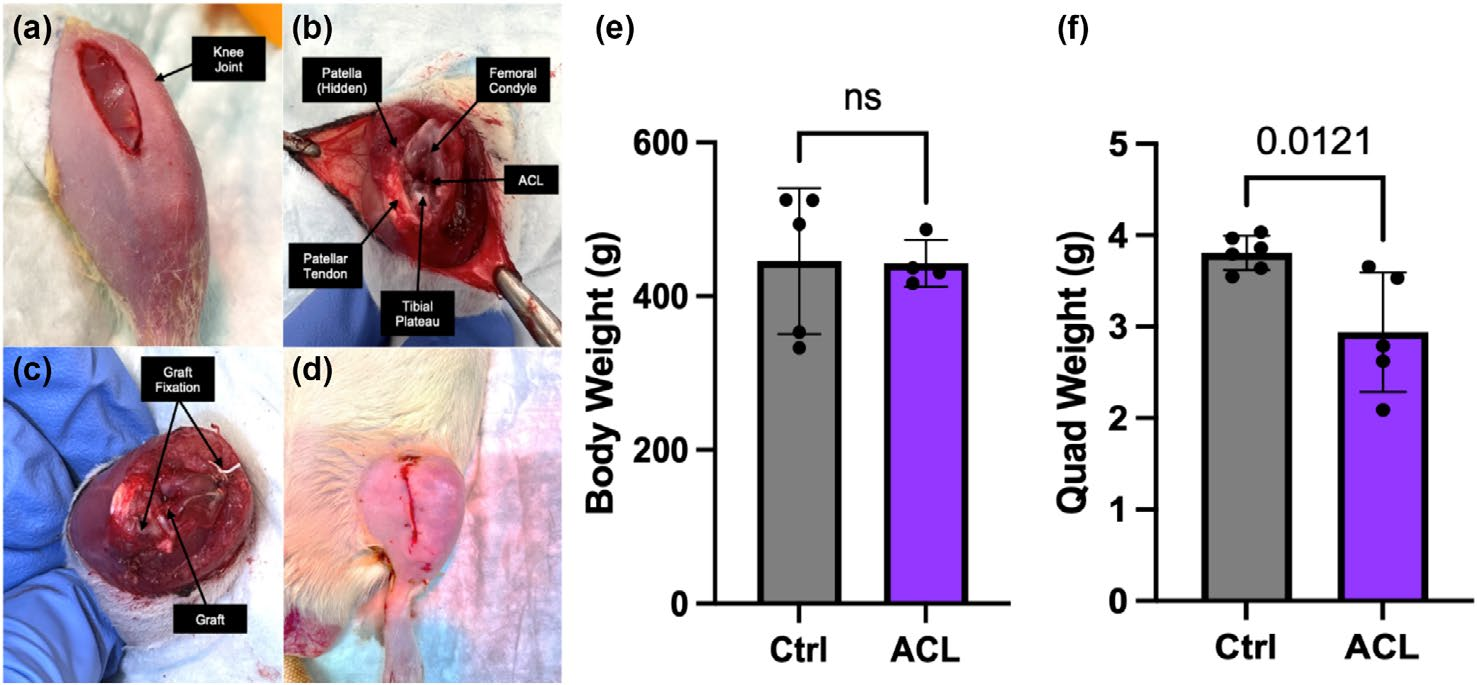
Muscle loss following bilateral ACL‐R in adult rats. (a) Skin incision on the hindlimb medial to the patella. (b) Visualization of knee joint capsule with the patella translated laterally to view joint structures. (c) Fixation of engineered allograft of the ACL. (d) Closure of the incision. (e) Bodyweight and (f) quadriceps weight normalized to body weight in nonsurgical and ACL‐R rats following a 2‐week recovery.

1. Prepare the surgical area with sterile draping over a heating pad.
2. Induce and maintain anesthesia by inhalation of 2% isoflurane with 2‐L/min oxygen (VetFlo, Kent Scientific, Torrington, CT) for the duration of surgery.
3. Inject subcutaneous analgesia: buprenorphine XR (3.25 mg/kg) and bupivacaine (5 mg/kg) and apply eye lubrication.
4. Remove fur from both hindlimbs (hip to ankle) using depilatory cream, and clean skin using aseptic technique.
5. Place sterile draping, leaving only the operative limb exposed.
6. With the knee in extension, make a 2‐cm long vertical incision with a scalpel medial to the patella, centered at the level of the patella (Figure 1a).
7. Retract skin laterally until the incision is centered over the knee joint. Slightly flex the knee and use the scalpel to open the parapatellar joint capsule by cutting medial to the patella and extending proximally to the level of the musculotendinous junction of the quadriceps and distally to the level of the patellar tendon insertion on the tibial tubercle. Avoid contact with the medial collateral ligament, patellar tendon, or quadriceps tendon.
8. With the knee flexed, use fine forceps to release and translate the patella laterally, exposing the intercondylar notch and femoral condyles (Figure 1b).
9. Transect the ACL in the intercondylar notch. Successful transection is confirmed by a positive anterior drawer test.
10. Using a power drill, place a 1.4–1.8‐mm stainless steel k‐wire on the ACL origin in the intercondylar notch and drill superolaterally through the lateral femoral condyle, exiting on the lateral aspect of the femur. Ensure a patent and adequately dilated bone tunnel by carefully drilling back and forth within the bone.
11. Thread a 1.4‐mm Keith needle (or tie a 3‐0 Vicryl suture) with one end of the suture tails of the graft. Pass the needle through the femoral tunnel, pulling one end through the bone.
12. Secure the tails of the graft using a needle driver or mosquito snap.
13. Place the K‐wire on the ACL insertion point on the tibial plateau and drill anteromedially to the anteromedial proximal tibia. The 1.4‐mm K‐wire is appropriate for rats ~300‐g and the 1.6‐mm or 1.8‐mm K‐wire for rats >400 g.
14. Thread the distal suture tails of the graft through the tibial tunnel. Secure the proximal suture tails on the femoral side with a snap to avoid displacing it as the distal end is pulled through the tibial tunnel.
15. With the knee in full extension, manually tension the graft and use a 4‐0 Vicryl suture to fix the femoral end of the graft to the surrounding periosteum with a figure‐of‐eight stitch. Similarly, secure the tibial end to the periosteum or soft tissue with a figure‐of‐eight stitch. Remove excess graft and suture, leaving 0.5–1 mm on each end past the securing stitch (Figure 1c).
16. Flush the joint capsule with saline. With the knee in full extension, reduce the patella and close the medial joint capsule securely with 4‐0 Vicryl or PGA suture.
17. Close the skin with 5‐0 Vicryl or PGA suture and seal the wound with wound glue (Vetbond, Japan) (Figure 1d).
18. Remove the draping and inject 500‐μL saline subcutaneously into the abdomen.
19. Place clean draping over the animal, exposing only the contralateral hindlimb, and repeat steps 5–17 on the contralateral hindlimb.
20. Inject 500‐μL analgesic (carprofen, 5 mg/kg) into the intraperitoneal (IP) space. Stop the anesthesia and remove the nose cone. Place the rat in a clean cage under fluorescent lighting. 5 mg/kg carprofen is also injected on postoperative days 1 and 2 for pain control.

### 2.2 Transient BFR‐exercise protocol

#### 2.2.1 Cuff placement protocol (day of BFR‐exercise intervention)

1. One day prior, anesthetize the rat and remove all fur from the hindlimb(s) using depilatory cream.
2. Rinse the skin with 70% ethanol or saline to reduce skin irritation.
3. Line the underside of the cuff with double‐sided tape.
4. Place the rat in the harness and secure the harnessed rat to the squat apparatus.
5. Ensure that the cuff is fully deflated and attached to the sphygmomanometer.
6. While gently restraining the rat manually, place the cuff around the proximal hindlimb, ensuring that it is secured above the knee.
7. Release the rat from restraint and watch for slipping or movement of the cuff, adjusting as necessary.

#### 2.2.2 BFR‐exercise protocol

1. Acclimation Protocol: 7–10 days: 1–2 days to freely roam with a clear box containing the squat apparatus for 30–60 min; 1–2 days to roam the space while wearing the harness for 30–60 min; 1–2 days strapped to the apparatus with no loaded weight for 7–10 min; 1–2 days in the apparatus with loaded weight <15% bodyweight; 1–2 days in the apparatus with loaded weight of 30% bodyweight. Acclimation can be incentivized with peanut butter.
2. Prior to the start of the BFR‐exercise protocol:
  - Load weights to the vertical pin until the desired load is reached. The lever arm weighs 80 g.
  - Acquire baseline blood/tissue samples prior to starting exercise.
3. Place the harness on the rat and tighten the straps.
4. Attach the rat to the squat apparatus. Adjust the “rest position” height to achieve full knee flexion. The front limbs of the rat should not contact the ground.
5. Attach the occlusion cuffs to the rat (see “Cuff Placement”).
6. Inflate cuffs to the therapeutic threshold (40%–80% of total limb occlusion pressure, ideally determined by ultrasound) using a handheld sphygmomanometer (see “Blood Flow Occlusion”).
7. Observe squat movement. Knee extension can be prompted (e.g., with tail shock, compressed air toward the rectum).
8. After completion of the desired repetitions, unfasten the rat from the apparatus, remove the cuff and harness, and return the animal to the cage.

Postsurgical animals should begin the BFR‐Exercise protocol no sooner than 2 weeks following surgery for adequate recovery.

#### 2.2.3 Muscle biopsy protocol

1. Day prior to procedure: Remove the fur of both hindlimbs from hip to ankle with depilatory cream; then cleanse the skin with 70% ethanol.
2. Day of procedure: Cleanse hind limb with 70% ethanol. Once dry, secure the rat in a prone position by gentle handheld restraint.
  - Consider use of a DecapiCone sleeve (Braintree Scientific, Braintree, MA) to gently immobilize the animal.
3. Anesthetize the skin of the hindlimb from the hip to the knee using topical lidocaine 5% cream.
4. Place the quadriceps in a shortened position by flexing the hip and extending the knee.
5. Use a scalpel to make a ≤3‐mm vertical incision of the skin over the vastus lateralis midway between the hip and knee joint. Carefully cut the overlaying connective tissue to visualize the muscle.
6. Orient a 2‐mm Nanobiter (Arthrex, Naples, Fl) over the muscle tissue. Quickly pinch and remove a small section of tissue; then retract the Nanobiter.
7. Clean and cover the wound with wound tape.

### 2.3 Blood flow occlusion imaging workflow

Functional ultrasound was performed to generate ultrasensitive 2D Power Doppler images of the rat hindlimb vasculature using the Iconeus One imaging system with a 2D linear ultrasound probe (25 × 17.5 × 6‐mm, 15‐MHz, 100‐μm spatial resolution, 1.5‐cm scanning depth, 128 elements) (Iconeus, Paris, France) (Macé et al., 2011). An anesthetized rat was transferred to a heat pad, and fur was removed from the hindlimb and abdomen with depilatory cream. The rat was positioned with the abdomen and hindlimb within range of the transducer. The hindlimb was extended while a 1.6‐cm digit cuff (Hokason Inc., Bellevue, WA) was secured above the knee. Ultrasound gel was applied inferior to the cuff to cover the entire imaging plane. The transducer was positioned above the hindlimb parallel to the femur such that the field of view extended superiorly to inferiorly in the axial direction of the hindlimb. The femoral artery and its bifurcation into the popliteal and saphenous arteries were visualized at a depth of 1–2 cm below the surface of the skin. Scans were acquired at a baseline without the cuff and over increasing external occlusion pressures. Screenshots of single fUS 2D scans were captured (SnippingTool, Windows) over an acquisition time of 120 s for each pressure. The diameter of the femoral artery was measured using Fiji software with the Diameter.class plugin (https://imagej.net/ij/plugins/diameter/index.html) (ImageJ).

### 2.4 Statistics

Graphpad Prism 10 was used for statistical analyses. Mean ± standard deviation is presented unless otherwise specified, and *p* < 0.05 was considered significantly different. Two groups were compared by the 2‐tailed unpaired Student's *t*‐test. Differences within animals were examined with a paired Student's *t*‐test. To evaluate the practical significance of observed differences, both effect sizes and percentage changes were calculated. Cohen's *d* were considered trivial, small, moderate, large, and very large when the values were 0–0.2, 0.2–0.6, 0.6–1.2, 1.2–2.0, and >2.0, respectively (Hopkins, 2006).

## 3 RESULTS

### 3.1 ACL reconstruction surgery as a model of postsurgical muscle atrophy in rats

Twenty young adult male Sprague–Dawley rats (aged 6–10 weeks) underwent bilateral ACL reconstruction surgery with a mean operative time from incision to closure of the surgical wounds of 40 min. There were no intra‐ or post‐operative complications. In the acute postsurgical period, the rats were partially weight‐bearing on the surgical hindlimbs but actively moving around the cage. By 1 week postsurgery, the rats were fully weight‐bearing with no limp. No wound abnormalities were observed. The rats displayed normal feeding, urinating, defecating, and sleeping postoperatively.

Rats were euthanized 14 days following surgery. At sacrifice, body weights of the control and ACL‐R rats were identical (Figure 1e), demonstrating tolerance of the procedure. Quadricep muscles were harvested bilaterally and weighed immediately after euthanasia. Normalized quadricep weight was lower in the ACL‐R rats compared to the non‐surgical control group. The effect size using Cohen's *d* was 0.868, indicating a large effect (Figure 1f). All data were normally distributed.

### 3.2 Generation and validation of blood flow restriction using functional ultrasound imaging

**FIGURE 2:**
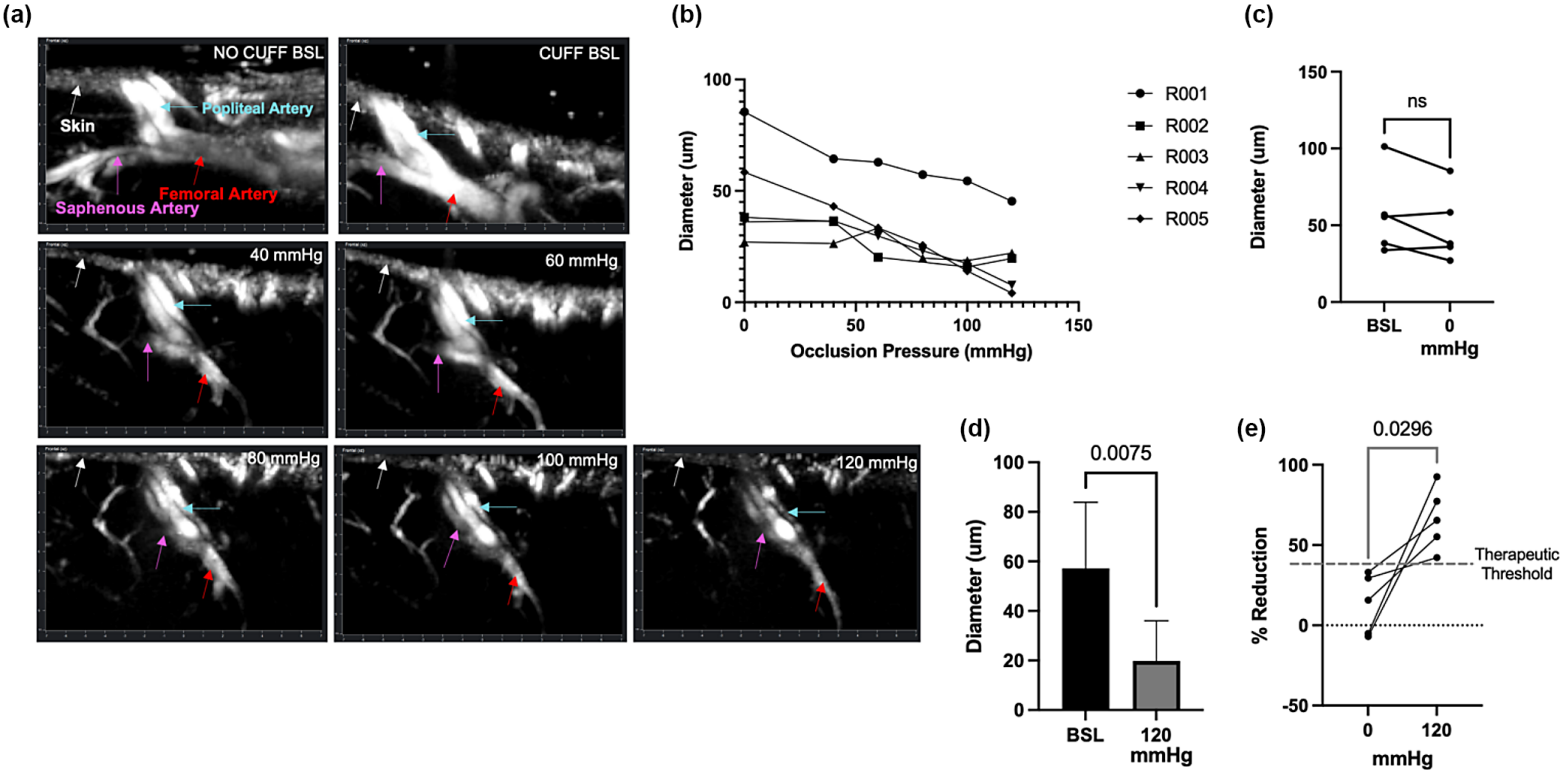
Functional ultrasound imaging of femoral artery with increasing external occlusion in rats. Diameter measured 1 mm from the bifurcation point on the femoral artery. (a) Representative 2D images acquired from a functional ultrasound scan of the femoral artery at the bifurcation point into the popliteal and saphenous arteries. Scans labeled with colored arrows: Skin‐white, femoral artery‐red, popliteal artery‐blue, saphenous artery‐pink. Images from a single rat. (b) Inter‐animal comparison of femoral artery diameters across increasing occlusion pressures. Scans were measured blindly. (c) BSL indicates the uncuffed baseline, and 0 mmHg the uninflated cuff baseline. Differences calculated using the paired Student's *t*‐test. (d) Mean diameter at no cuff baseline (BSL) versus inflated to 120 mmHg. (e) Percent reduction of femoral artery diameter from BSL at 0 mmHg and 120 mmHg.

Given its size and length compared to the body size, the femoral artery was used to generate and validate blood flow restriction (Figure S1a–c). Reduction of femoral artery diameter was observed with increasing external cuff pressures (Figure 2a,b). There was no difference in mean diameter between an uncuffed (BSL) and cuffed (0‐mmHg) baseline, despite inter‐animal variability (Figure 2c). Percent reduction in femoral artery diameter was 67% at 120 mmHg (Figure 2d, Table 1). The effect size using Cohen's *d* was 1.5, indicating a large effect. At 120 mmHg, femoral artery diameter reduced to a therapeutic range for muscular adaptations to BFR (>40%) in all rats (Figure 2e). Skin changes, including swelling and erythema, were present during cuff inflation, but normalized within 1 min after cuff deflation. After awakening, the animals did not show any signs of pain or functional deficits.

**TABLE 1:** Change in femoral artery diameter with increasing external occlusion. Occlusion pressures measured in mmHg. Each value represents change from baseline femoral artery diameter in rats without the digital cuff.

Rat	% change from no cuff baseline					
	0 (cuff)	40	60	80	100	120
1	15.6	36.4	38	43.4	46.2	55.2
2	33.1	36.5	64.6	–	72.7	65.4
3	29.4	31.2	12.9	48	51.2	42.2
4	−6.92	−8.02	12	31.5	48.5	77.3
5	−5.02	22.5	40	54.1	74.8	92.6

### 3.3 Weighted hindlimb knee extensions to model BFR‐exercise in the rat

**FIGURE 3:**
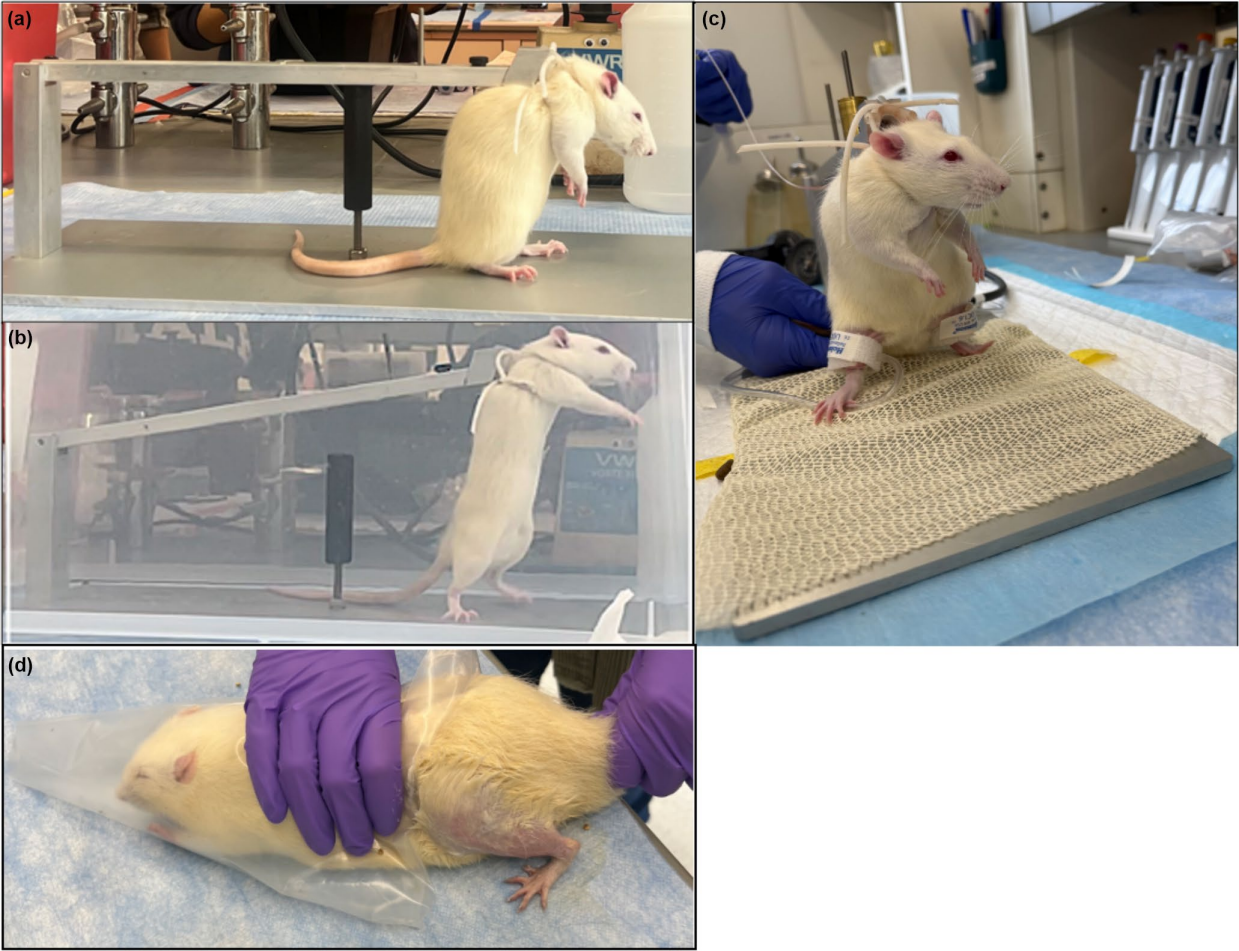
Resistance exercise model. (a) Representative photo of the rodent secured onto the squat apparatus in the ‘rest’ position (full knee flexion). (b) Rat in the apparatus in a full knee extension position. (c) Rat in the apparatus with bilateral cuffs on the hindlimbs. (d) Gentle hand restraint of rat placed in Decapicone sleeve to immobilize while exposing hindlimb for biopsy.

Ten rats (aged 6–10 weeks) were used. Figure 3a demonstrates day 4 of the acclimation during which the rat freely explores the squat apparatus while wearing the harness. Rats achieve full knee flexion at rest (Figure 3b) and full knee extension (Figure 3c) to complete one full repetition. Loaded resistance up to 30% of the rat bodyweight was achieved by adding weighted washers to the 80 g arm of the squat apparatus.

### 3.4 Adaptation of 2 mm Nanobiter to facilitate muscle biopsies in an awake rat

Rats were situated in a Decapicone sleeve for immobilization (Figure 3d). The 2 mm Nanobiter was well tolerated following topical 5% lidocaine application. Tissue samples yielded 5–15 mg per sampling. No biopsy site abnormalities or functional deficits were observed post biopsy.

## 4 DISCUSSION

A suitable animal model of BFR‐exercise is crucial for a comprehensive understanding of its underlying physiologic mechanisms. Here, we describe a comprehensive protocol establishing a rat model of BFR‐exercise in a postsurgical rehabilitative setting.

Here, we surgically transect and reconstruct the ACLs of rat hindlimbs bilaterally in a single procedure using a commercially available copolymer graft. The use of engineered co‐polymer grafts to re‐establish ligament function in rat ACL‐R surgery has been previously described with varying efficacy (Bashur et al., 2009; Freeman et al., 2007; Gisselfält et al., 2002; Kawakami et al., 2021; Leong et al., 2014, 2015; Seo et al., 2009). FlexBand is approved for use in humans to augment soft tissue reconstruction procedures (Myers et al., 2022; Peterson et al., 2014). To our knowledge, the FlexBand has not been used as an ACL replacement in rats. Here, we show successful integration and recapitulation of the functional properties of the ACL in the operative limbs using the FlexBand. We also show ~26% reduction in quadriceps weight 2 weeks following ACL‐R in rats, demonstrating that ACL‐R is a viable model of muscle loss in rats. Considering that sarcopenia is an independent predictor of a 37% increase in mortality risk across studies in elderly people and individuals with cancer, cardiovascular, liver, lung, and renal diseases (Koon‐Yee Lee et al., 2021), there is an urgent need for new models that recapitulate acute muscle loss and which can be used to test therapeutic modalities.

Although ACL tears and subsequent reconstructive surgery most commonly occur unilaterally in humans, we perform bilateral ACL transection and reconstruction to best integrate into studies of BFR‐exercise in awake rats. This avoids potentially confounding compensation from the nonsurgical contralateral leg during metabolic or physiologic analyses.

Generating transient BFR in rats to induce muscle hypertrophy and strengthening during resistance exercise contrasts more permanent methods of vascular restriction used to model vascular pathology, such as femoral and/or saphenous artery ligation. Models of intermittent hindlimb ischemia exist to improve physiology and outcomes in humans and have been modeled in animals (Ali et al., 2007; Murry et al., 1986). For example, ischemic preconditioning followed by periods of reperfusion to a region distant from the heart uses intermittent ischemia to occlude arterial inflow to protect against prolonged ischemia (Ali et al., 2007). Compared to BFR, ischemic preconditioning involves 20 min of complete occlusion via direct arterial clamping, which must be performed by a trained surgeon and doubles the duration of BFR. From a vascular perspective, BFR is distinct from other methods of intermittent limb ischemia given its noninvasive and accessible nature.

Functional ultrasound imaging (fUSI) is highly advantageous to evaluate cerebrovascular physiology and pathophysiology in humans and animals (Demene et al., 2017; Demené et al., 2021; Dizeux et al., 2019; Imbault et al., 2017; Sieu et al., 2015). The high sensitivity of the fUSI allows for visualization of the microcirculation in response to perturbation. Its ease of use, quantification capabilities, and improved resolution compared to conventional Doppler ultrasound imaging allow for a standardized workflow to validate BFR in animals.

To determine the occlusion pressures necessary to generate therapeutic BFR in rats, we used the Iconeus fUSI system to directly visualize and measure the femoral artery in vivo. To our knowledge, this is the first study to utilize functional ultrasound for validation of BFR in a rat model. We observed reductions in vessel diameter at 80 and 120 mmHg within the therapeutic range for muscular adaptation (DePhillipo et al., 2018). This method directly translates to clinical protocols of BFR to the lower limb in humans. Compared to other studies using external cuffs to interrogate vascular changes, our adaptation of the fUSI system allows for more direct and precise quantification of BFR in rats. Our degree of occlusion is consistent with other measurements of BFR. Yoshikawa et al. and Tanaka et al. used the same external occlusion cuff as presented in this report to evaluate the effects of BFR on muscle growth in neuromuscular electro‐stimulated plantar pedis and soleus muscles of anesthetized rats (Tanaka et al., 2021; Yoshikawa et al., 2019). The groups achieved vessel occlusion within the therapeutic range for muscle adaptation, but it is not specified which vessels were probed.

When generating this model, the fUSI system presented a few challenges. First, while the animals are anesthetized during imaging, abdominal movement during respiration caused slight shifting of the transducer during acquisition, affecting resolution quality. Thus, we immobilized the pelvis and hindlimb by hand restraint. Second, physiologic differences in size and orientation of the vasculature in each animal made identification challenging. Variability in animal position and measurement necessitated varying exposures to anesthesia, which may have caused vascular changes (Akata & Warltier, 2007).

To establish BFR‐exercise in an awake rat, we found that the 1.6‐cm cuff is appropriate for the upper hindlimb of adult rats as small as ~300–400 g. To secure and stabilize the cuff during in vivo resistance exercise experiments, we applied the cuff lined with a thin strip of double‐sided tape after removing the fur from the hindlimb. This allows for targeted induction and maintenance of BFR in an awake, exercising rat.

Tail‐weighted ladder climbing and incline treadmill walking do not directly replicate the clinical use of BFR‐exercise in rehabilitation settings. We describe the use of a squat apparatus (adapted from Tamaki et al.) (Tamaki et al., 1992), which allows for isolated full knee flexion and extension. Other groups have also adapted this apparatus to model resistance exercise in rats (Miguel‐dos‐Santos et al., 2021; Barretti et al., 2017; Stefani et al., 2018). However, there has not been a direct application of this model in combination with BFR. We configured a flexible harness to the machine both for optimal rodent comfort and the ability to access vascular lines for experimental infusions or sampling. Other studies using this apparatus also detail a training protocol that includes distinct sets and repetitions (e.g., 4 sets of 12 repetitions) (Barretti et al., 2017). Finding this schema difficult to enforce, we extended our acclimation period as needed for each animal. Second, we prompted knee extension with compressed air targeted to the rectum to encourage full range of motion movements and reduce stress responses. Third, we counted repetitions based on the quality of movement (e.g., full knee extension and return to full knee flexion as one repetition). This method allows for variability in the number of sets and repetitions and/or the duration of exercise.

To facilitate longitudinal analysis of the intramuscular environment in response to BFR‐exercise, we developed, to our knowledge, the first method for repeated in vivo muscle tissue sampling that maintains full functionality in a rat. We used a 2 mm Nanobiter to manually harvest muscle tissue from the lateral quadriceps. However, we attempted other biopsy apparatuses with varying efficacy. First, we tested a 22 G × 11.0 cm spring‐loaded biopsy needle (Tenmo A.C.T., Merit Medical, Jordan, Utah). This system required a 20G introducer with a coaxial connector hub. This method was invasive, produced excessive discomfort, and could not be completed repeatedly. Further, it yielded an inadequate <1 mg of tissue. Next, we replicated the suction‐assisted Bergstrom muscle biopsy system used in humans (Shanely et al., 2014). We scaled this system down to include a 1‐mm cored needle, an outer trocar, and a connected syringe for use in our rat model (Figure S2). Tissue yields were larger, but inconsistent (~2–9 mg muscle tissue). Despite these challenges, we found that the rats tolerate the procedure with 5% topical lidocaine. This adapted suction‐assisted cored needle biopsy system can be effective for repeated muscle biopsies in rats.

Despite these strengths, several weaknesses exist. First, these experiments include relatively small sample sizes. However, the bilateral ACL‐R and BFR validation experiments showed large magnitudes of effect and reliability. Second, our BFR and exercise model may induce a stress response, which can affect the tolerability of the intervention and confound metabolic and behavioral outputs. Lastly, our bilateral ACL‐R model requires the use of a synthetic allograft of the ACL, which does not exactly mimic an autograft. The impact of these factors will require a more comprehensive analysis in future studies.

In conclusion, we demonstrate a comprehensive, clinically relevant model of BFR‐exercise. These methods allow for mechanistic and physiologic studies of BFR, resistance exercise, or combined BFR‐exercise.

## AUTHOR CONTRIBUTIONS

A.E.F. and R.J.P. were involved in conceptualization. A.E.F., S.C.B.R.N., C.A., and C.T. were involved in methodology. A.E.F., S.C.B.R.N., I.C.M.; Y.O., H.B., and A.D. were involved in investigation. A.E.F., C.T. and R.J.P. were involved writing, review and editing. R.J.P. was involved in funding acquisition and resources. R.J.P. and C.A. were involved in supervision.

## FUNDING INFORMATION

A.F. received support from CTSA Grant Number UL1 TR001863 from the National Center for Advancing Translational Science (NCATS), a component of the National Institutes of Health (NIH). This work was supported in part by National Institutes of Health (National Cancer Institute) award R37CA258261 (to R.J.P.). Its contents are solely the responsibility of the authors and do not necessarily represent the official views of NIH.

## CONFLICT OF INTEREST STATEMENT

None.

## ETHICS STATEMENT

All animal experiments were carried out according to protocols approved by the Yale University Institutional Animal Care and Use Committee (protocol 2022‐20290).

## Supporting information


Figures S1–S2.

## Data Availability

All original data are found in the Supplemental Materials.
