# Supplementary material for: A comprehensive model of blood flow restriction in the postsurgical rat
Source: Physiol Rep. 2025 Aug 5;13(15):e70495. doi: 10.14814/phy2.70495 (PMC12325096; doi:10.14814/phy2.70495)
Supplement: Supplementary file 1 — Figures S1–S2. [file PHY2-13-e70495-s001.pdf]

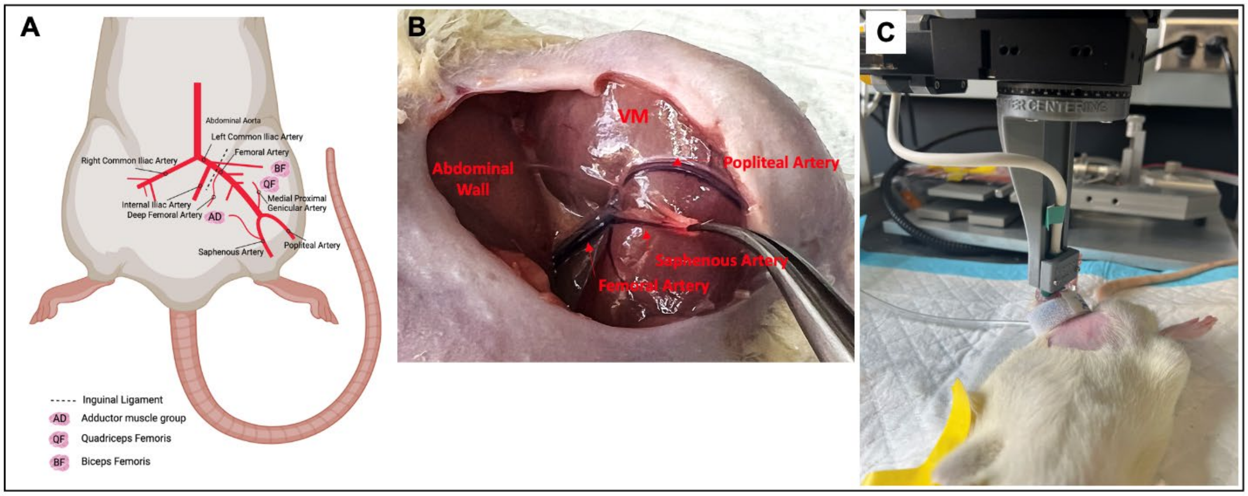

Supplementary Figure 1. Anatomic and visual presentation of BFR modeling in rat. (A) Rat hindlimb vasculature schematic depicting placement of ultrasound probe adapted from Aref, Zeen et al. *Intl J Molec Sci*. 2019. Created in BioRender. (B) Dissection of rat hindlimb to visualize probed vessels. Anatomic sites labeled in red text. VL = vastus medialis (C) Depiction of functional ultrasound transducer positioned on the rodent hindlimb over the femoral artery distal to the external occlusion cuff.

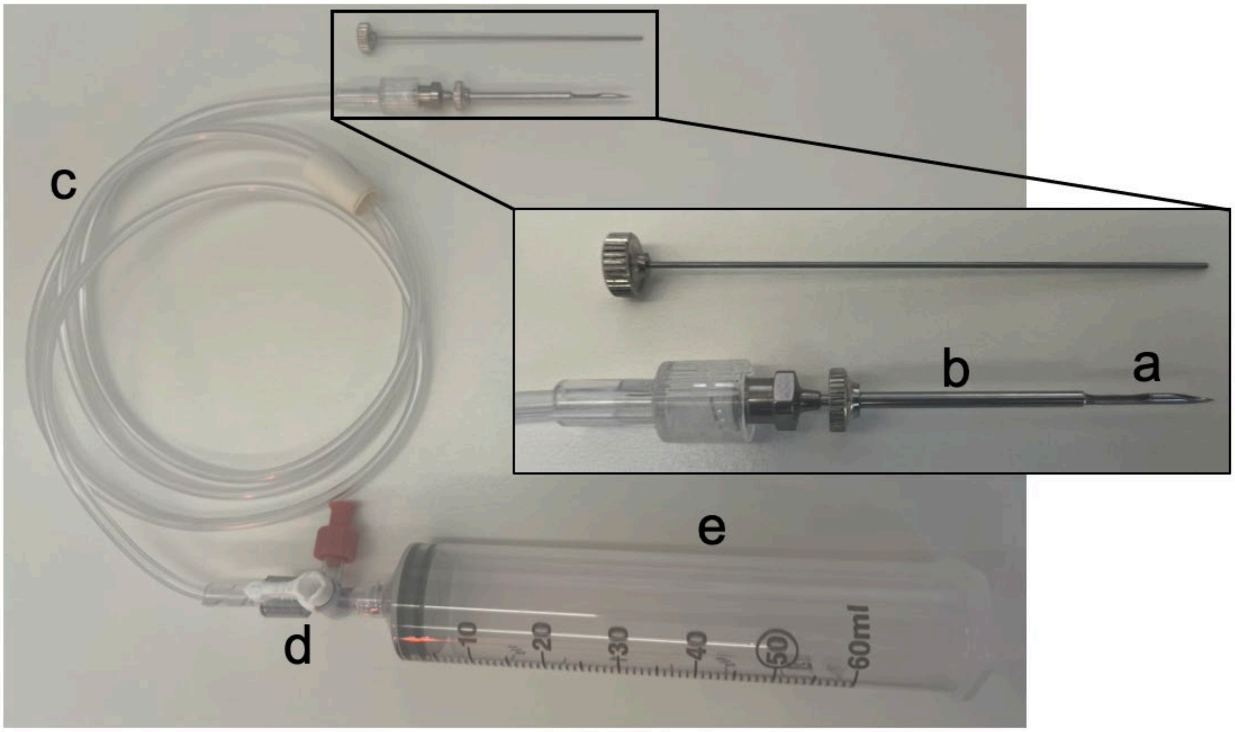

Supplementary Figure 2. Adapted suction-assisted cored-needle biopsy system for repeated muscle biopsy in the rat. Suction-assisted cored-needle biopsy system adapted from the Bergstrom muscle biopsy apparatus, which includes a (a) 1mm cored needle, (b) outer trocar, (c) plastic tubing, (d) three-way stop-cock, and (e) 60mL syringe.

Figure 1 Raw Data

#	Group	BW(g)	L Leg. (g)	R Leg. (g)	Avg	Normalization Factor	Quad Weight (norm)	% of BW
1	ACL	646.3	2.85	5.48	4.165	1.232221163	3.380075043	0.64443757
2	CTRL	455.2	3.19	3.33	3.26	0.867874166	3.756304921	0.71616872
3	ACL	430.5	2.72	3.08	2.9	0.820781697	3.533217189	0.67363531
4	ACL	416.7	2.86	2.95	2.905	0.794470925	3.656521478	0.69714423
5	ACL	436.8	1.53	2.84	2.185	0.832793136	2.623700778	0.50022894
6	ACL	486.8	2.08	1.8	1.94	0.928122021	2.090242399	0.39852095
7	CTRL	524.5	4.01	3.71	3.86	1	3.86	0.73593899
8	CTRL	493.5	3.87	3.72	3.795	0.940896092	4.033389058	0.76899696
9	CTRL	525.2	3.6	4	3.8	1.001334604	3.794935263	0.72353389
10	CTRL	353.2	2.7	2.2	2.45	0.673403241	3.638236127	0.69365798
11	CTRL	332.7	2	2.5	2.25	0.634318398	3.547114518	0.67628494
12	CTRL	399	3.01	3.03	3.02	0.7607245	3.969899749	0.75689223
13	ACL	441.7	2.2	2.5	2.35	0.842135367	2.790525243	0.53203532
14	ACL	413.8	2.8	2.9	2.85	0.788941849	3.612433543	0.68873852

Figure 2 Raw Data

Rat 1

Occlusion Pressure	Diam1							
0 (no cuff)	-132.0417	-132.14503	-132.31651	-132.33717	-132.85101	132.338284		
0 (no cuff)	-126.37235	-126.7689	-127.30679	-128.35382	-130.6813	127.896632		
0 (no cuff)	-99.11894	-97.89127	-96.15907	-95.30132	-94.92907	96.679934		
0 (no cuff)	-90.82343	-85.22223	-86.13213	-87.22142	-88.22397	87.524636		
0 (no cuff)	-62.610085	-62.64214	-62.16844	-61.315346	-60.97495	61.9421922	101.2763356	
0 (cuff)	-58.717056	-57.92008	-55.927876	-54.731045	-53.951767	56.2495648		
0 (cuff)	-114.49839	-115.52302	-115.04624	-114.77522	-115.48318	115.065209		
0 (cuff)	-86.11568	-87.10454	-85.70692	-83.75582	-82.17903	84.972398	85.42905727	15.64756295
40	-54.429066	-55.25807	-55.11827	-54.782402	-55.31989	54.9815396		
40	-76.637146	-77.36524	-78.83141	-80.8947	-80.46133	78.8379652		
40	-58.653538	-58.77149	-59.835716	-60.575535	-59.05833	59.3789218	64.39947553	36.41211925
60	-59.814663	-60.623383	-62.147495	-64.27221	-67.34951	62.8414522	62.8414522	37.95050759
80	-41.32858	-43.253246	-43.380272	-42.963776	-42.87422	42.7600188		
80	-61.13169	-60.87083	-61.668007	-61.990425	-62.830475	61.6982854		
80	-68.59062	-65.492615	-65.76926	-67.91446	-69.725815	67.498554	57.31895273	43.40340972
100	-45.759758	-45.536346	-45.4673	-44.424892	-43.431072	44.9238736		
100	-57.42019	-61.06683	-62.18318	-62.96891	-63.61011	61.449844		
100	-55.581738	-56.96524	-57.669506	-57.897217	-56.49591	56.9219222	54.43187993	46.25409817
120	-38.87863	-39.251503	-39.101006	-39.42812	-53.79241	42.0903338		
120	-31.06916	-30.33185	-31.636274	-32.82792	-32.498043	31.6726494		

Rat 2

0 (no cuff)	-49.62151	-48.684486	-51.761127	-51.02078	-50.18994	50.2555686		
0 (no cuff)	-55.705524	-57.093636	-61.337555	-64.79351	-66.541725	61.09439		
0 (no cuff)	-53.112064	-52.915283	-51.963097	-51.49443	-53.600597	52.6170942		
0 (no cuff)	-62.16513	-63.37224	-64.55617	-64.84912	-64.90932	63.970396	56.9843622	
0 (cuff)	-53.27677	-53.340275	-53.19289	-50.334774	-50.085674	52.0460766		
0 (cuff)	-25.821861	-25.70816	-30.151157	-29.17893	-28.095575	27.7911366		
0 (cuff)	-40.28365	-41.78875	-33.762894	-33.848972	-35.609318	37.0587168		
0 (cuff)	-31.335602	-35.355713	-27.773361	-29.14756	-29.294483	30.5813438	38.11191728	33.1186385
40	-44.436356	-43.87165	-43.39509	-42.067947	-41.64052	43.0823126		
40	-32.05837	-31.95384	-31.00129	-30.18448	-28.594023	30.7584006		
40	-19.239178	-19.461943	-20.059774	-20.219007	-19.596066	19.7151936		
40	-40.70683	-40.76938	-41.18567	-42.30295	-42.58718	41.510402		
40	-48.570446	-49.330585	-47.18461	-41.801754	-41.98719	45.774917	36.16824516	36.52952536
60	-14.996147	-16.500637	-15.939552	-15.241832	-14.076943	15.3510222		
60								
60	-13.440147	-12.918269	-14.063862	-15.000642	-13.806066	13.8457972		
60	-33.02358	-32.513115	-33.24913	-32.139168	-25.60129	31.3052566	20.16735867	64.60895957
80								
80								
80								
80								
80								
100	-20.877827	-21.442953	-13.794523	-25.097427	-25.938805	21.430307		
100	-9.909214	-9.10725	-10.069475	-12.150005	-10.096127	10.2664142	15.8483606	72.18822851
120	-13.557452	-13.061319	-13.049213	-13.162925	-12.874363	13.1410544		
120								
120	-26.42376	-27.042816	-26.27107	-25.911602	-25.584307	26.246711	19.6938827	65.43984711

Rat 3

0 (no cuff)	-49.65491	-49.465385	-49.918354	-49.659748	-48.606472	49.4609738		
0 (no cuff)	-39.048176	-39.556133	-39.84032	-39.92725	-40.411453	39.7566664		
0 (no cuff)	-39.925262	-30.833359	-30.100485	-36.5829	-37.10285	34.9089712		
0 (no cuff)	-28.39472	-28.669302	-29.17314	-29.238005	-29.014086	28.8978506	38.2561155	
0 (cuff)	-24.515732	-24.842125	-24.822397	-24.656353	-24.781631	24.7236476		
0 (cuff)	-31.458475	-28.320698	-27.104713	-25.964207	-26.857658	27.9411502		
0 (cuff)	-20.744822	-21.09278	-22.404524	-23.069967	-23.704205	22.2032596		
0 (cuff)	-34.45072	-34.824833	-32.564167	-31.759811	-32.37039	33.1939842	27.0155104	29.38250513
40	-24.968718	-24.724567	-23.929102	-23.248922	-23.170246	24.008311		
40	-45.104424	-26.391483	-42.421055	-20.573647	-30.742653	33.0466524		
40	-22.300398	-22.588898	-22.407738	-22.813337	-21.035069	22.229088		
40	-20.979164	-20.655415	-21.120462	-29.500172	-30.372726	24.5255878		
40	-27.207846	-27.273516	-28.134113	-28.424906	-28.347822	27.8776406	26.33745596	31.1549131
60	-36.12555	-35.588535	-34.028084	-25.866402	-26.671217	31.6559576		
60	-39.41241	-29.709389	-30.063263	-30.623188	-33.000626	32.5617752		
60	-36.692215	-35.322906	-33.975594	-32.19012	-31.440636	33.9242942		
60	-36.13279	-36.689064	-38.28948	-38.772198	-40.486042	38.0739148		
60	-33.454277	-31.546364	-30.28228	-29.020008	-27.737244	30.4080346	33.32479528	12.89027952
60								
80	-24.54591	-24.660229	-24.433775	-24.870098	-25.073242	24.7166508		
80	-26.430702	-26.650373	-27.946945	-26.096508	-17.556496	24.9362048		
80	5.9677067	8.066227	9.750693	12.422424	13.793963	10.00020274		
80							19.88435278	48.02307417
100	-17.45986	-17.397259	-18.097855	-17.97374	-18.719173	17.9295774		
100	-17.762144	-19.298586	-19.878613	-19.721819	-20.523046	19.4368416		
100								
100							18.6832095	51.16281605
120	-20.4691	-19.06168	-20.70679	-23.92492	-26.328852	22.0982684		
120								
120							22.0982684	42.23598473

Rat 4

0 (no cuff)	-40.368893	-40.433113	-40.164314	-40.48375	-40.101627	40.3103394		
0 (no cuff)	-36.40045	-30.073145	-33.621452	-35.667446	-34.435276	34.0395538		
0 (no cuff)	-25.42293	-25.524025	-27.61589	-28.373745	-28.167553	27.0208286	33.7902406	
0 (cuff)	-38.23468	-36.422203	-38.90091	-40.07163	-39.45619	38.6171226		
0 (cuff)	-34.68714	-35.78163	-33.791668	-34.460857	-34.34793	34.613845		
0 (cuff)	-38.332195	-37.016296	-33.558746	-33.60185	-32.39063	34.9799434		
0 (cuff)	-33.626858	-37.066734	-34.861916	-37.791866	-38.05067	36.2796088	36.12262995	-6.902553248
40	-40.74727	-40.441227	-39.00449	-38.89507	-35.744713	38.966554		
40	-37.662586	-37.871853	-40.972157	-40.0843	-39.78266	39.2747112		
40	-26.744743	-28.263584	-30.268444	-29.884567	-30.59143	29.1505536		
40	-41.098454	-33.340633	-33.1215	-40.33551	-45.17222	38.6136634	36.50137055	-8.023411204
60	-25.131325	-25.985212	-25.695833	-25.188576	-25.370602	25.4743096		
60	-36.205235	-38.265312	-36.293076	-36.53721	-35.64532	36.5892306		
60	-32.054436	-29.701233	-26.491816	-24.180418	-23.164484	27.1184774	29.7273392	12.02389012
80	-18.071651	-17.42751	-16.816076	-16.32227	-23.173246	18.3621506		
80	28.956425	28.031696	27.434761	27.172043	28.12899	27.944783		
80							23.1534668	31.47883416
100	-15.401677	-21.146622	-13.199806	-12.3168	-15.563883	15.5257576		
100	-22.160833	-22.357693	-21.670912	-20.751566	-19.923708	21.3729424		
100	-16.725649	-15.43011	-14.190606	-15.548059	-14.754946	15.3298739	17.40952463	48.47765413
120	-7.536751	-5.162037	-9.352972	-8.810486	-7.5666103	7.68577126		
120								
120							7.68577126	77.25446424

Rat 5

0 (no cuff)	-32.616474	-31.943012	-32.56788	-31.962067	-32.452805	32.3084476		
0 (no cuff)	-61.869583	-62.523006	-63.51981	-63.280724	-65.49315	63.3372546		
0 (no cuff)	-64.13207	-64.155685	-64.398605	-66.74313	-68.78999	65.643896		
0 (no cuff)	-58.904804	-61.06965	-61.48303	-61.472626	-62.52993	61.092008	55.59540155	
0 (cuff)	-59.87867	-56.21463	-58.726906	-59.529934	-60.833683	59.0367646		
0 (cuff)	-60.07542	-61.652187	-61.54243	-59.09614	-58.479008	60.169037		
0 (cuff)	-62.04278	-61.463585	-60.846325	-59.890335	-59.211903	60.6909856		
0 (cuff)	-52.41909	-52.18907	-54.77595	-54.615814	-54.26972	53.6539288	58.387679	-5.022497135
40	-48.777466	-49.079803	-47.81189	-47.16093	-46.859222	47.9378622		
40	-20.89013	-27.048386	-44.616394	-48.618443	-18.911604	32.0169914		
40	-50.221573	-50.53956	-48.834007	-48.202652	-48.17273	49.1941044	43.04965267	22.56616291
60	-25.088947	-23.69421	-22.37658	-47.942818	-47.76905	33.374321		
60								
60							33.374321	39.96927791
80	-22.474028	-32.382893	-24.386606	-23.215073	-25.138643	25.5194486		
80								
80								
80							25.5194486	54.09791478
100	-15.901279	4.928238	-15.131149	-22.671452	-21.403055	14.0357394		
100								
100							14.0357394	74.75377638
120	5.025008	4.0775585	3.719901	3.7428179	3.996297	4.11231648		
120								
120							4.11231648	92.60313557
